# Minimal Requirements for the Emergence of Learned Signaling

**DOI:** 10.1111/cogs.12351

**Published:** 2016-03-14

**Authors:** Matthew Spike, Kevin Stadler, Simon Kirby, Kenny Smith

**Affiliations:** ^1^ Language Evolution and Computation Research UnitSchool of Philosophy, Psychology & Language SciencesUniversity of Edinburgh

**Keywords:** Communication, Cultural evolution, Signaling games, Reinforcement learning, Feedback learning, Observational learning, Agent‐based models, Exemplar theory

## Abstract

The emergence of signaling systems has been observed in numerous experimental and real‐world contexts, but there is no consensus on which (if any) shared mechanisms underlie such phenomena. A number of explanatory mechanisms have been proposed within several disciplines, all of which have been instantiated as credible working models. However, they are usually framed as being mutually incompatible. Using an exemplar‐based framework, we replicate these models in a minimal configuration which allows us to directly compare them. This reveals that the development of optimal signaling is driven by similar mechanisms in each model, which leads us to propose three requirements for the emergence of conventional signaling. These are the creation and transmission of referential information, a systemic bias against ambiguity, and finally some form of information loss. Considering this, we then discuss some implications for theoretical and experimental approaches to the emergence of learned communication.

## 1 Introduction

Human language provides a uniquely flexible and expressive system of communication, but we are not the only species capable of communication. Signaling behavior is found throughout nature: Virtually every species has a means of communicating about, for example, the presence of food or predators, potential as a mate, or presence of a competitor. However, only human communication displays such *open‐endedness* (Tomasello, 2010) in the number of signals learned, the contexts in which they are elicited, and the responses they effect. This flexibility arises because the basic building blocks of human language—words—are learned socially, by observing word use by others. In contrast, the articulatory form of signals in the vast majority of animal communication systems are, as far as we know, not socially learned. For instance, among our closest relatives, the form of alarm calls (distinctive calls used to warn conspecifics of the presence of particular types of predators) are thought to be largely genetically determined (Fedurek & Slocombe, 2011); even among the other apes, while the decision to employ a given call may be intentional (Slocombe et al., 2010), there is only limited evidence for any flexibility or group‐level variation in the form of those calls (Crockford, Herbinger, Vigilant, & Boesch, 2004; Seyfarth & Cheney, 1986). There are of course obvious exceptions; for instance, many bird species are capable of both learning and innovating songs (Podos, Huber, & Taft, 2004). However, vocal learning in birds (and in other animals where it has been observed, such as cetaceans, elephants, and bats: Janik, 2014; Poole, Tyack, Stoeger‐Horwath, & Watwood, 2005; Boughman, 1998) is most probably a case of convergent evolution, rather than reflecting some ancestral cognitive capacities shared by the extremely distant common ancestor of all vocal learning species.

Innately specified communication systems are presumably the product of natural selection (Maynard Smith & Harper, 2003). As such, the major questions concern the nature of the evolutionary route to signaling, as well as the selective pressures involved. The emergence of *learned* communication, on the other hand, is less well understood. First, we might ask when and why a learned system would replace an innately specified signaling system (Lachlan, Janik, & Slater, 2004; Ritchie & Kirby, 2006). Second, socially learned communication systems are potentially shaped by an entirely different set of pressures. In a learned communication system, unlike its innate equivalent, natural selection cannot directly tune the structure of the signaling system; rather, socially learned signaling systems are shaped by the processes through which they are learned and used (see literature review in next section). Their functional properties are then determined by the nature of the learning and usage mechanisms involved (which are themselves potential targets for biological evolution). Understanding the nature of these mechanisms is crucial to understanding when and how a learned communication system such as human language might evolve.

Our focus in this study is therefore: What are the necessary social and psychological adaptations which allow populations to develop, via processes of learning and use, functional learned communication systems? Semiotic experiments (such as surveyed by Galantucci & Garrod, 2011) demonstrate that human subjects can rapidly bootstrap communicative conventions across a range of modalities and interactive conditions. Moving beyond the laboratory, the recent emergence of indigenous sign languages (e.g., Nicaraguan Sign Language and Al‐Sayyid Bedouin Sign Language: Senghas, Senghas, & Pyers, 2005; Sandler, Meir, Padden, & Aronoff, 2005) is a compelling reminder that functional communication systems are able to *self‐organize* in human populations in the absence of any explicit, centralized coordination. Presumably, the same mechanisms also underlie the development of other human signaling conventions, including all other human languages. Identifying these mechanisms—which must be particular to human cognition and interaction—will shed light on what enables *Homo sapiens* to be such a fundamentally communicative species.

The emergence of functional learned communication has been studied across a number of seemingly loosely related disciplines, including Classical and Evolutionary Game Theory (e.g., Lewis, 1969; Nowak, Krakauer, & Kingdom, 1999; Skyrms, 2010), Artificial Life (e.g., Steels & Loetzsch, 2012), Cognitive Science (e.g., Barr, 2004), and Evolutionary Linguistics (e.g., Oliphant, 1996; Smith, 2002). The assumptions made and conclusions drawn in these various fields regarding the prerequisites for functional communication appear on the surface to be quite different, if not mutually incompatible. To cut through a rather confusing mesh of approaches, models, and results, we have created a framework to replicate a representative selection of the approaches outlined above. Having done this, we then identify a *basic framework*—an urn‐model—which strips the individual models back to the simplest set of common underlying mechanics.

We then employ an *additive* approach to this framework: We first add the characteristic features of each model in terms of *interaction* and *learning*. None of these basic instantiations reliably lead to optimality; as such, we then investigate which particular *mechanisms* are responsible for doing so. By adding each mechanism in isolation, we are able to investigate exactly which are responsible for driving the behavior of each model. The subsequent direct comparison reveals that the apparent diversity of mechanisms driving the emergence of functional learned communication is overstated in the literature; in fact, the same fundamental processes underpin *all* of the current accounts.

The rest of this study is organized as follows. In Section 2 we discuss the issues of conventionality and optimality, and how these are tackled in models drawn from the various disciplines mentioned above. We first motivate and then describe the exemplar‐style framework which we have used for our model replications in Section 3, before discussing each replication in more detail in Section 4, along with the adjustments we have made for comparison, and which aspects of each model are necessary for the development of optimal communication. In Section 5 we propose that three fundamental principles—the creation and propagation of referential information, a bias against ambiguity, and a mechanism leading to information loss—determine whether any system is able to bootstrap functional communication. Finally, this leads into a discussion in Section 6 regarding how this can help us interpret the various theories of the emergence of communication outlined above.

## 2 Past approaches to the emergence of functional learned signaling

For any form of communication to be functional, it must be *conventional*; in particular, there must be consensus within a population about how signals are produced and interpreted. Conventions are widespread in human populations and extend far beyond the communicative domain (we have, for instance, conventions about what to wear to work, what side of the road to drive on to get to work, what times one should work at, and appropriate language at work).

In his classic study, Lewis (1969) analyzes convention as a type of game‐theoretic *coordination problem*: Two or more agents have a choice of behaviors, and coordinating those behaviors leads to mutual benefit. Even working under the assumption that such coordination provides a mutual benefit, the mechanisms leading to the establishment of conventions are not immediately obvious. Lewis proposed a critical role for *common knowledge* (Lewis, 1969; p. 56): All agents are aware of a set of propositions, each agent knows that every other agent also knows those propositions, and so on recursively *ad infinitum*. Populations of agents can employ this knowledge to create conventions by making rational choices targeting maximal individual payoffs. However, in the case of these *simple* conventions (where an atomic choice is made from an unordered set of alternative behaviors, such as the side of the road we drive on), Vylder (2008) shows that such sophisticated reasoning is unnecessary: Whenever agents *strongly amplify* observed behavior, population‐wide agreement on a single convention is assured. (This is where agents sample from each other's behavior, and the suite of behaviors is represented as a ranked probability distribution. In strongly amplified copying, the ratio of likelihoods between any two subsequently ranked behaviors is strictly increased in favor of the more highly ranked one.)^1^

However, being conventional is not enough to ensure a functional communication system. Lewis's Signaling Game (1969) is the canonical problem in the emergence of learned communication. In its most basic form, the Signaling Game involves a single signaler and a single hearer. The signaler must communicate one of two possible world‐states to the hearer with two available signals, and the hearer has a choice of two possible responses. Each world‐state has a corresponding “matched” response which triggers a mutual payoff; mismatched responses provide no payoff. Lewis showed that even this simple game has several *Nash equilibria*, where a Nash equilibrium is any state where the best payoff for any given player is to continue with her current strategy, leading to a global stasis where no further change in play can occur. Only two of these Nash equilibria are *optimal* strategies, designated by Lewis as signaling systems, which guarantee that the hearer will select the appropriate response based only on the signaler's signal. The others—*pooling equilibria*—are stable but non‐optimal strategies; for example, if the signaler sends the same uninformative signal for every world‐state and the receiver always chooses the action with the greatest average payoff. This ambiguity of signal‐to‐response mapping will be referred to below as *homonymy*.

**Figure 1:**
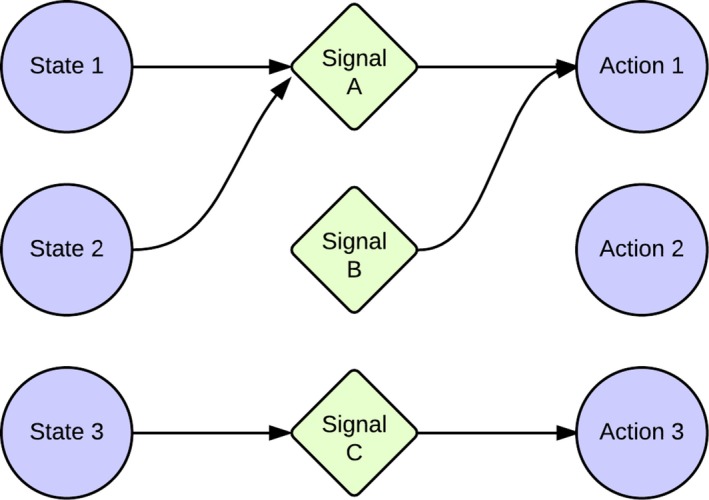
An example of a *partial pooling equilibrium*. The first two states, signals and actions, are *pooled* and mutually uninformative: The speaker always produces the Signal A and the receiver always uses Action 1. State 3, however, leads to the informative Signal C and hence Action 3.

Moving beyond the simplest scenario of two world‐states, two signals, and two responses results in a drastic increase in the number of possible system states, and the chance of multiple *partial* pooling equilibria: These are stable states that are a mixture of informative strategies and pooled, non‐informative ones (see Fig. 1). As such, in addition to being conventional, a functional communication system must be at least somewhat informative; in Lewis's terms, it must allow the hearer to select the correct response with greater than chance frequency. The most functional systems are optimal; in Lewis's terms, such systems require that all world‐states must map to at least one signal, and each of those signals must be unambiguously associated with a matched response. Identifying how conventional, optimal, learned signaling systems develop has therefore become the benchmark problem in this field; the existence of many non‐optimal stable states in the face of Lewis's rational behavior suggests that the reliable development of optimal signaling occurs via some other means. In the sections that follow, we review the proposals made in various fields as to what that mechanism might be.

### 2.1 Payoff‐based accounts

Game‐theoretic accounts are driven by the idea of a *payoff*, which is instantiated through either increased evolutionary fitness (e.g., Nowak et al., 1999) or reinforcement learning (e.g., Skyrms, 2010), where individuals modify their behavior in response to the payoffs they receive.

Nowak et al.'s model involves the *natural selection of cultural variants* (Boyd & Richerson, 1985) and rests on two assumptions: First, the fitness of individuals (i.e., the number of their offspring) is determined by their communicative success within a population, and second, the resulting children learn from their parents (with some error), thereby inheriting their communication system via social learning. Numerical simulations show that, under these conditions, while some populations evolve optimal systems, many stabilize at partial pooling equilibria, some signals being associated with more than one meaning. However, these suboptimal states occur less as the chance of learning error increases: Error knocks the systems out of previously stable states. Note that the mechanism at play here—natural selection—is the same as that invoked to explain the evolution of signaling systems which are not socially learned, the only difference being that the mechanism through which behaviors are inherited is social learning, rather than genetic transmission.

Skyrms (2010) surveys a type of *reinforcement learning* devised by Roth and Erev (1995). Many forms of reinforcement learning (e.g., Bush & Mosteller, 1953) are essentially “memoryless”: Each available behavior can be completely characterized by a single (probabilistic) value which describes its current state. The effect of any learning experience recalculates this value using a parameterized function. Roth–Erev reinforcement, on the other hand, models memory as a collection of tokens which gradually accumulate over a learner's lifetime; as such, calculating both behavior and the effects of learning must take into account not just the relative proportion of memory tokens, but their absolute count as well (experiences in later life contribute relatively little to the overall store of tokens, whereas early experiences have larger effects). Skyrms (2010) motivates the Roth–Erev model by showing that learners in a non‐signaling scenario—one where they must learn to modify their behavior to maximize their expected returns when presented with an initially unknown distribution of world‐states—are able to escape pooling equilibria by using Roth–Erev reinforcement learning, whereas parametric forms of reinforcement learning (Bush & Mosteller, 1953) are not. Incorporating this into the basic Lewis signaling game, Skyrms (2010) describes a simple iterative strategy: Each time a pair of agents successfully communicate, the associations involved are strengthened by both. A proof of convergence to optimality using Roth–Erev learning in the minimal signaling game (two world‐states, signals and responses) is given in Beggs (2005). However, Barrett (2006) shows that including more states, signals, or responses immediately increases the possibility of non‐optimal equilibria, and that simple reinforcement no longer leads to guaranteed convergence on optimal signaling. Barrett (2006) goes on to propose two solutions to this problem: the addition of negative reinforcement (also known as *punishment*, the term we shall use henceforth), where unsuccessful associations are decremented, and *forgetting* (which is further investigated in Barrett & Zollman, 2009). Both strategies greatly increase the likelihood that optimal signaling develops and guarantee it for certain parameter regimes.

Also using Roth–Erev reinforcement (but looking at pragmatic implicatures rather than signaling games), Franke and Jäger (2012) investigate the effects of *lateral inhibition*: After successful communication, competing associations are dampened. With this effect included, they show via simulation that optimal states are reached far more quickly. We draw attention to the role of lateral inhibition here, as it plays an important factor in several other models described below.

### 2.2 Interaction‐based accounts

The program of artificial life research as set out in Steels (2012) and the neural network populations of Barr (2004) place a critical emphasis on the fundamental roles of *feedback* and *alignment*. Agents interact with each other multi‐modally: Repeated attempts at local alignment ultimately lead to a globally functioning communication system.

As part of a larger program to investigate the evolution of language, Steels (2012) shows that multi‐modal negotiations between embodied robotic agents situated in a complex environment lead to the development of multiple levels of language‐like structures. The seminal *Naming Game* described in Steels and Loetzsch (2012) is a core element in this process. A population of agents is situated in an environment containing a number of objects. Pairs of randomly chosen agents are presented with a limited context of objects observable by both parties, with one particular target object chosen for a designated signaler to attempt to communicate to the hearer. The agents then execute a scripted series of actions. A signal is sent, the hearer's interpretation is checked, and the intended referent is indicated by the speaker in the event of failure. Both agents then potentially adjust their internal representations by strengthening and weakening associations, with these weight adjustments determined according to the particular scenario (success or failure) which has just occurred—these updates can be carried out by speaker, hearer, or both agents involved in the interaction. In addition, agents possess the ability to *innovate* terms for previously unseen objects, chosen from a very large signal space. This is a potentially critical difference with the other models discussed in this section, which assume limited signal spaces, and in which processes which eliminate homonymy are critical to establishing optimal signaling. In contrast, in a typical Naming Game simulation involves an initial stage in which the number of terms for any given object explodes, before a single term wins out for each, as the result of gradual *lateral inhibition* of competing terms. A consequence is that homonymy is very rare: “optimality” for these games tends to be defined not in terms of successful communication, but by when the lexicon is reduced to a minimal size. De Vylder and Tuyls (2006) show that, as shown with simple conventions in Vylder (2008), convergence on a minimal, unambiguous, conventional lexicon is guaranteed if agents utilize a *strongly amplifying* imitation function (as described in Section 2). Baronchelli (2010) further shows that hearer update (i.e., hearers updating their internal representations based on the success or failure of an interaction) is critical in the development of optimal lexicons, while speaker update plays a lesser role.

Barr (2004) looks at the role of common knowledge in the emergence of conventional communication. Employing populations of interacting agents (both neural network based and simpler association based), he showed that not only was common knowledge (about the signaling behavior of the population as a whole) unnecessary, but that population‐wide convergence on a single system was significantly more likely when agents used *only* the information from individual interactions. The neural network model is rather sophisticated, but it includes a type of parametric reinforcement learning similar to Bush and Mosteller (1953) (outlined in Section 2.1). Also included is a form of lateral inhibition, (although described as a *mutual exclusivity bias*) which acts to promote one‐to‐one signal/meaning mappings. Barr's simulations reliably lead to states of optimal signaling: In a second set of results, Barr aims to counter a possible objection to his neural networks: as they sample over time from the whole population, they could be argued to be accruing a type of common knowledge. To this end, he uses a modified association‐based model (based on Steels, 1997) which employs a “*stay/switch*” strategy—agents stick to successful strategies with some chance of switching to less successful ones. This model includes a type of memory where agents can be restricted to knowledge of their last *n* interactions. In the end, both types of population, neural network and stay/switch, reliably arrived at global convergence on an optimal system.^2^ A further observation was that stay/switch populations proved more efficient at developing globally optimal signaling when their memories were highly restricted, providing another strong counterexample to common knowledge‐based explanations.

#### 2.2.1 Reinforcement versus feedback learning: An aside

It is worth clarifying the differences between reinforcement and feedback accounts, as they actually share much in common. Another complicating point is that the models in Barr (2004) and, to a lesser extent, Steels and Loetzsch (2012) are described at different times in terms of both Reinforcement and Feedback learning.

One of the main factors distinguishing reinforcement (in its classic form) and feedback involves the availability of *referential information*. This describes how agents associate meanings with signals, both for when signals are sent and interpreted. In fact, in classic signaling games, referential information is irrelevant. Mutually available “meanings” are split into two: world‐states perceivable by the speaker, and actions taken by the receiver. However, as every state has a single matched action which triggers a payoff event, we can (for the sake of direct comparison) temporarily overlook this distinction and see matched state/action pairs as directly equivalent to meanings in the other models. In any case, in reinforcement accounts the equivalent of referential information is only made available after a successful interaction, and it is provided by the *environment*: Signaler and receiver know that the intended and interpreted meaning have coincided, because they receive reinforcement from the environment; more subtly, in the event of failure the *absence* of positive reinforcement informs each party that his or her choice has been unsuccessful. In feedback learning, on the other hand, the environment cannot provide this information. Instead, the agents themselves must furnish it via “pointing” behavior: Simple social interactions, presumably via another modality, which are able to resolve reference. As such, although there is a near equivalent to the reinforcement described above, it is analyzed in terms of the interaction between the agents; the receiver must point at its interpreted referent, providing *Interpretation Feedback*, and the speaker must either indicate whether the receiver has selected the correct meaning (what we shall term *Yes/No Feedback*) or provide richer information by indicating its intended referent (henceforth *Referential Feedback*). With Yes/No Feedback, then, the situation resembles reinforcement learning in that full referential information is only made available after communicative success. The real difference between reinforcement learning and Yes/No Feedback learning is seen after failure. In Reinforcement learning, the speaker can only know that his or her intended signal/meaning association was unsuccessful. Similarly, the hearer is only aware that his or her interpreted association failed. This is also true for Referential Feedback, but due to the availability of Interpretation Feedback, extra information (about how the hearer interpreted the signal) is reliably available to the speaker. In Reinforcement learning this information is only available after successful communication. Referential Feedback plays a similar role, as it provides full information about the speaker's intended referent to the hearer; again, with reinforcement learning this is only available after success.

It is the availability of this extra information in feedback learning that allows for more subtle strategies than in reinforcement learning. With Reinforcement learning, agents must somehow promote successful associations and inhibit failed ones. This remains the case with Feedback learning, but speakers and hearers have reliable sources of referential meaning which are *independent* of communicative success. How this information is used, of course, depends on the particular model.

Interestingly, then, although Barr (2004) describes what must be a feedback model—in that alignment is verified through interaction—the interaction itself is placed in a black box. Because of this, the model uses an exact equivalent to the reinforcement dynamic: The extra information potentially available is not actually used. In models such as that of Steels and Loetzsch (2012), the interaction has a more fine‐grained realization which is incorporated into the model, making the extra sources of information potentially usable. The question, then, is to determine what role those extra sources of information *do* play; this will be dealt with in Section 4.

### 2.3 Observational learning accounts

A third strand of work has focused on the evolution of communication via *iterated learning* (Kirby, 2001) — repeated cycles of production and observational learning, often but not always with population turnover. In generational turnover models, one generation of learners learns from behavior produced by the previous generation of learners and goes on to produce behavior which is observed and learned from by a subsequent generation of learners; alternatively, new agents acquire a signaling system by observing the existing population produce and/or interpret signals, then replace an older member of the population, implementing a gradual turnover of the population. This *observational learning* paradigm typically de‐emphasizes the role of communicative interaction (see e.g., Oliphant, 1996; Smith, 2002): Agents are assumed to be unmodified by any further interaction after an initial phase of learning, and signaling conventions can therefore only develop during this initial stage of sampling and learning. For this reason, the models place a critical emphasis on the learning process itself. Furthermore, unlike the reinforcement and interaction‐based models discussed above, these observational learning models typically do not include any referential uncertainty: Learners learn from observing meaning‐signal pairs, rather than signals produced in some context which leaves its intended meaning unclear.

The models in Smith (2002) investigate how individual *learning biases* shape the evolution of signaling systems in populations through iterated learning. In this study, learners are modeled as simple associative networks, who adjust association weights after each learning exposure according to a particular learning rule: Smith varies these learning rules parametrically, to explore both the properties of learning at the individual level and the consequences of these individual‐level processes for the signaling systems which develop in populations. Smith used three criteria to classify the effect of each learning rule: whether it produced agents capable of (a) *learning*, (b) *maintaining,* and (c) *constructing* optimal signaling systems; each criterion is a strict subset of the previous one. A property shared by all constructor‐type rules is an implicit bias against homonymy in the form of lateral inhibition. Learners with such a bias are less likely to successfully learn homonymous meaning‐signal mappings, and over many episodes of learning this bias eliminates homonymy entirely, leading to optimal signaling. Learning rules which are neutral to homonymy are sometimes capable of constructing functional signaling, but usually converge on suboptimal pooling equilibria. In contrast, biases against synonymy alone do not contribute toward the development of optimal systems, although they are required for the learning of optimal systems under certain assumptions about the relative size of the meaning and signal spaces (K. Smith, 2004).

Oliphant and Batali (1996) adopt an alternative approach within the observational learning framework, exploring a rational approach which they dub *obverter*. Their work starts from the observation that the rational approach to signaling is to maximize the chances of being correctly understood, while rational receivers will attempt to maximize the chance of correct interpretation. Obverter signalers leverage this fact by calculating which signal is most likely to be correctly interpreted as their intended meaning, based on the observed reception behavior of the population; similarly, obverter reception involves identifying which meaning is most likely to be signaled using the received signal, again based on observations of the population's production behavior. Oliphant and Batali first show that when agents have perfect information about the signaling behavior of the population (e.g., through unlimited observation of the production and reception behavior of that population), the communicative accuracy of a population will necessarily increase with every new generation of learners who apply the obverter approach to production and reception, eventually leading to convergence on an optimal system. Numerical simulations show that *approximating* this perfect knowledge, by estimating the population's signaling behavior from a limited number of observations of population behavior, is sufficient to guarantee optimal communication.

### 2.4 Summary

What conclusions can we draw from the above? First, there are clearly substantial differences between the various models (see Table 1 for a brief summary of their key features): What is striking is their heterogeneity, with each model having at least one unique feature, and no obvious universally shared property which might drive the evolution of functional signaling systems. Second, the models differ in the time‐scales involved. Some accounts employ intergenerational learning and natural selection, and describe a process which takes place over multiple generations: Selective reproduction or language acquisition is the only mechanism of change. In contrast, in other models, signaling systems are negotiated between individuals over much shorter time periods. Finally, and most important from our perspective, the various models seem to require rather different cognitive capacities in individual agents. Skyrms' (and subsequently Barrett's) reinforcement learners have no social or cognitive capacity beyond the capacity to retain a set of signal/meaning association weights and the ability to recognize a payoff. Nowak's evolutionary model excludes even the requirement for individuals to recognize communicative success, leaving natural selection to do the work of tuning the population's communication system. In contrast, the cognitive apparatus required in the Naming Game and Observational Learning paradigms seems rather more demanding—they variously require mechanisms of speaker–hearer feedback (often glossed in these models as “pointing”), various processes of competition or lateral inhibition between signals and meanings, rational reasoning about the optimal signaling behavior, and possibly the ability to reliably infer a signaler's intended meaning. However, drawing strong conclusions as to the necessary cognitive prerequisites for the emergence of functional learned signaling seems premature, since the numerous implementational differences between the existing models potentially obscure a common underlying mechanism.

**Table 1:** A comparison of the major features of the models surveyed in Section 2

	Nowak	Steels	Barrett/Franke	Oliphant/Batali	Smith	Barr
Transmission	Vertical	Horizontal	Horizontal	Vertical	Vertical	Horizontal
Model Type	Association matrix	Associative	Numerical	Associative	Neural	Neural
Mod. Hearer/Speaker?	H & S	H & S	H & S	H	H	H & S
Interaction	Mutual payoff	Feedback	Mutual payoff	Observation	Observation	Mutual payoff
Features	Natural selection	Inhibition	Frget/pun/inhib	Obverter	Inhibition	Inhibition
Production/Reception	Stochastic	Deterministic	Stochastic	Deterministic	Deterministic	Deterministic

At this point some notions require clarification. In our simulations reported below, our key criterion will be whether a particular type of model develops *optimal signaling*—a completely unambiguous set of signals which cover all meanings—*reliably*, that is, 100% of the time. Human lexicons are not optimal but are supported by contextual cues to disambiguate words which would be ambiguous out of context (e.g., Piantadosi, Tily, & Gibson, 2012); Why, then, are we looking for reliable optimality when it does not appear in natural language? The first reason is largely historical: There is a long tradition in work looking at the evolution of signaling conventions to focus on the evolution of optimal signaling, with the development of signaling systems which are near‐optimal in context being a more complex (and under‐explored) question. Second, on a practical point, the work which follows demonstrates that models which do not *always* produce optimal systems actually very rarely do so, to an extent that would be highly dysfunctional in human language.

## 3 An exemplar‐based framework

To cut through this diversity of models, we have replicated four of the six models above using a minimal framework.^3^ The two which have been excluded are Nowak et al. (1999) and Barr (2004). The former is excluded simply because the mechanism driving the development of optimal signaling is the well‐understood process of natural selection, simply operating on traits which are inherited culturally rather than genetically; as such, this work has relatively little to say about how the processes of learning and use might shape signaling systems. Barr (2004) is not replicated primarily because, unlike the other models reviewed above and presumably driven by his neural network implementation, meanings and signals are not atomic, but are represented as distributed patterns of activation across both input and output nodes. This feature is hard to reconcile with the other models presented here. However, as can be seen in Table 1, Barr employs a mixed design, incorporating reinforcement and lateral inhibition. Since these features can be seen independently in one or more of the other models, we hope to derive insights into these processes in isolation which will also apply to Barr's model.

We employ a simple *exemplar*‐based “urn model” as a general framework for a number of theoretical and practical reasons. Although the replicated models involve several different forms of representation, all of them treat a signaling system as a set of associations between meanings and signals. The exemplar model captures this simply—meaning/signal pairs can be seen as “meaning” balls in “signal” urns (or vice versa): Mechanisms of learning and adjustment are equivalent to adding and removing the balls from the specified urn. This also allows for an unlimited number of novel signals in the same manner as Steels and Loetzsch (2012), unlike the fixed‐size neural networks of, for example, Smith (2002). In addition, this representation is identical to the reinforcement learning of Roth and Erev (1995). Roth–Erev learning directly captures the first two of exemplar theory's “central notions of similarity, frequency, and recency” (Walsh, Möbius, Wade, and Schütze 2010), p.1. The third factor is more problematic, as all stored tokens in Roth–Erev learning are equally weighted. This factor means that Roth–Erev learning is a simplified exemplar model. With that said, recency effects are introduced by including *forgetting*, such as in Barrett and Zollman (2009). As such, the exemplar framework can directly replicate game‐theoretic work such as Skyrms (2010) and Barrett (2006), as well as be easily extended to include the core mechanisms from feedback and observational learning accounts.

In the sections that follow, we describe the various components of our exemplar framework, before identifying a basic framework, a baseline instantiation of our model which includes the minimal ingredients to replicate the various results from the literature. In Section 4 we show these replications and explore various deviations from the minimal model which illuminate the fundamental mechanisms driving the evolution of optimal signaling.

### 3.1 Exemplars, agents, populations

Discrete meanings and signals *m, s* are drawn from unordered sets *M, S*. Each exemplar represents a simple bidirectional association between a single meaning *m* and signal *s*. Exemplars are atomic and unweighted. The population contains a set of agents *A*; each agent *a* consists of an unstructured set of *N* exemplars. The current number of exemplars of agent *a* associating meaning *m* with signal *s* is denoted by *N*_ams_.

We assume that populations are fully connected: Each agent has an equal chance of interacting with any other. During an interaction, two agents are chosen (the particulars of this depend on the *population dynamic*, which is described in Section 3.1.5 below), and designated *speaker* and *hearer,* respectively. A *context C ⊆ M* of *c* meanings is selected with uniform probability. Also with uniform probability, a single *topic* meaning *t* is selected from *C*. The speaker produces an *utterance u ∈ S* associated with the topic which the hearer turns into an *interpretation i ∈ M*. Production and interpretation can be described as stochastic functions over probability distributions for signal production *p*(*u|t*) and reception *r*(*i|u, C)*, where *C* is a context containing the topic *t*. The different ways in which exemplar storage gives rise to these probability distributions is specified below.

#### 3.1.1 Production and reception

**Figure 2:**
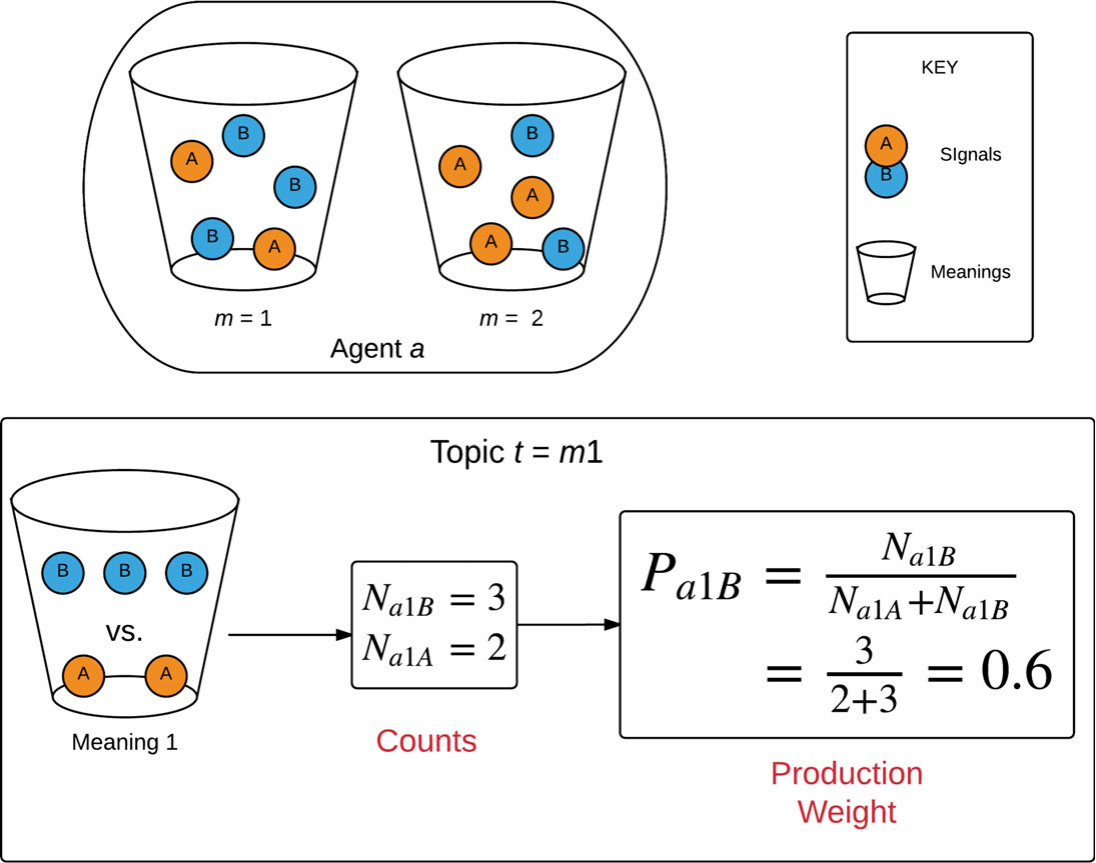
An *urn‐model* conceptualization of the notation for exemplar counts and production weights. Signal/meaning associations are represented as balls (signals) in urns (meanings). The weighting function simply counts the proportion of a given ball/signal in the urn/meaning chosen as topic, in this case meaning 1. For this example, WTA production would always produce signal B, whereas stochastic production would produce it 60% of the time. Although this diagram represents production weights P*_ams_*, visualizing the reception weights R*_amsC_* simply involves swapping the roles of signal and meaning to urns and balls, respectively, excluding any meanings which are not in the context *C*.

During production, given a target meaning *t*, an agent *a* must select a signal. In all the models we consider, each potential signal *s ∈ S* has a weight proportional to the proportion of stored exemplars which feature topic *t* paired with *s*. As shown in Fig. 2, in the standard model the *production weight* of a signal *s* is simply:

(1) $$P_{\mathrm{ats}}=\frac{N_{\mathrm{ats}}}{\sum_{s^{\prime }\in S}N_{{\mathrm{ats}}^{\prime }}}$$

We can similarly define the reception weighting of a potential interpretation *i* ∈ *M,* given an utterance *u* and a *context C* of potential meanings to which *u* could refer:

(2) $$R_{\mathrm{amuC}}=\frac{N_{\mathrm{amu}}}{\sum_{m^{\prime }\in C}N_{{\mathrm{am}}^{\prime }s}}$$

To select a signal based on *P,* or a meaning based on *R*, an agent could apply either a *stochastic* or *winner‐take‐all* procedure. A stochastic signaler (or receiver) simply samples a meaning (or signal) proportional to *P* (or *R*), that is, *p*(*u|t*) *∝ P*_atu_, *r*(*m|u, C) ∝ R*_amuC_. A signaler applying a winner‐take‐all procedure selects with uniform probability among the set of meanings (or signals, during reception) with the maximum value of *P* (*R* for reception); that is,

(3) $$p\left(s|m\right)=\left\{\begin{array}{cc} 1/|S^{\prime }| & \text{if}\;s\in S^{\prime }\\ 0 & \text{otherwise} \end{array}\right.$$

where S′ is the set of meanings for which *P*_amu_ is at a maximum, and *r*(*m|u, C)* is similarly defined with respect to those signals for which *R*_amuC_ is at a maximum.

The *obverter* mechanic differs from these standard production and reception procedures in that the obverter weights for production are simply equivalent to the reception weights in the standard model, and vice versa, that is, *P*_obverter_ **= *R*,* R*_obverter_ **= *P*.

#### 3.1.2 Communicative accuracy

Successful communication occurs when the hearer's interpretation matches the speaker's intended meaning, the topic (i.e., *t *= *i*). The communicative accuracy between a speaker *a* and hearer *b*, where *T* is the set of all contexts of size *c* is^4^:

(4) $$CA\left(a,b\right)=\frac{1}{C\cdot |T|}\sum_{C\in T}\sum_{m\in C}\sum_{s\in S}p\left(s|m,C\right)\times r\left(m|s,C\right)$$

The communicative accuracy of a population is the mean value after Eq. (4) is calculated for all possible pairs of agents in the population, with each member acting as both speaker and hearer.

#### 3.1.3 Learning, deletion, inhibition, and memory

*Learning* always involves storing exemplars—after an interaction one or more meaning‐signal pairs is added to an agent's memory. Memory limitations are modeled by enforcing a maximum allowed number of stored exemplars per agent. When this is surpassed, any newly stored exemplar results in the deletion of a randomly selected older exemplar.

Some models also involve deletion of exemplars, for example, in some reinforcement models to adjust the speaker and/or hearer's memories after an unsuccessful interaction. *Simple deletion* removes a single exemplar with a specific meaning‐signal association. *Lateral inhibition* represents competition between newly introduced exemplars and already stored ones. This can be either *anti‐homonymy*, which deletes exemplars which share the same signal as a given focal exemplar but a different meaning, or *anti‐synonymy* for the converse process. Lateral inhibition can be either *minimal*,* broad*, or *maximal* inhibition. Minimal inhibition deletes only a single competing exemplar, selected with uniform probability from all competing exemplars. Broad inhibition affects all competing associations equally: A single exemplar is deleted for each competing type. In maximal inhibition, *all* competing tokens are removed. When this method is applied against homonyms and synonyms, it guarantees that all signal/meaning mappings will be one‐to‐one. This instantly removes the problem of how signals become unambiguous (although not the problem of how populations arrive upon a shared system), and as such we do not investigate this mechanism here.

In all the cases where lateral inhibition proves necessary, minimal inhibition is sufficient: As such, we use minimal inhibition in all the replications, which shows that stronger forms are not necessary for the evolution of optimal signaling.

#### 3.1.4 Feedback and referential information

**Figure 3:**
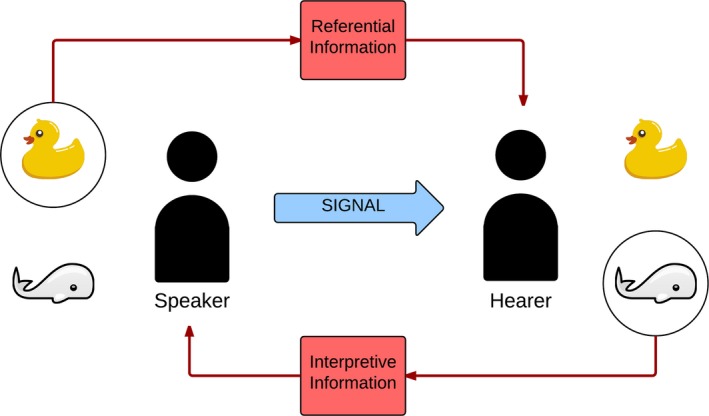
*Feedback*: the referential elements that can make up the feedback found in the different models: Speakers select a meaning and produce a signal, which is interpreted by the hearer. Information about the intended and interpreted referent is, depending on the model, always, sometimes, or never transmitted.

“Feedback” is not defined consistently across the literature, but it always describes a scenario in which referential information is transmitted *after* signaling (see Fig. 3). This may be about either the speaker's intended referent (*referential information)* or the hearer's interpretation *(interpretive information)*. Further to this, feedback can be either *full* (the referent is unambiguously indicated) or *partial* (some information about the referent is supplied, by limiting the set of candidates). Depending on the particular model, neither, one, or both of the elements in may be involved.

In reinforcement learning, full referential and interpretive information is provided after successful communication (i.e., the environmental payoff indicates to the speaker that the hearer's interpretation was identical to the topic, and this indicates to the hearer that its interpretation was identical to the speaker's intended meaning); the absence of payoff from an unsuccessful communication provides partial referential and interpretive information (the signaler knows that the receiver's interpretation was not the topic; the receiver knows that the topic was some meaning m∈C,m≠i). Observational Learning models provide full referential information: The hearer receives the topic plus the speaker's utterance.

Finally, several flavors of feedback are provided in the Naming Game, as discussed in Section 2.2.1. *Interpretation feedback* is when full interpretive information is provided (i.e., the hearer points at its interpretation). We again note that the interaction in the Naming Game requires that this information is always provided. With *Yes/No Feedback*, full referential information is provided after successful interpretation, but only partial referential information otherwise (i.e., after the hearer points, the speaker says yes or no). A richer alternative to this is *Referential Feedback*: Full referential information is provided (if the hearer is correct, the speaker confirms; if not, the speaker points at its intended referent).

#### 3.1.5 Population dynamics

The population can be either *closed* or exhibit *gradual replacement*. Closed populations have no turnover: No agent leaves the population, no new individuals enter the population, and learning occurs after each interaction, such that all members of a population continue to learn over the entire run of the model. Under gradual replacement, old agents are continually replaced with new ones; a new agent is created and interacts with the established population a given number of times before joining the population by replacing the oldest agent. In this scenario, following the standard Observational Learning paradigm, agents only learn during their initial set of interactions, on entering the population, and do not learn in their subsequent interactions (when they are serving as language model for some other new individual).

In both cases, the populations are fully connected: In the closed dynamic, each interaction involves a new pair of agents selected at random from the population; with intergenerational turnover, the new learner is paired with a randomly selected member of the established population.

### 3.2 The basic framework

We first establish the *basic framework*. This represents what we have identified as the simplest abstraction of the models surveyed in Section 2. The purpose of this is twofold: First, by providing a common framework, we can meaningfully compare the different models. Second, and more important, we can apply the various mechanisms from those models to *build up* from the basic framework. This *additive* approach allows us to see, both individually and (if necessary) in combination, the effects of each mechanism and ultimately determine which factors are responsible for the development of optimal learned signaling.

The basic framework is a type of Roth–Erev “urn” model. Agents are represented as a collection of “meaning/signal” tokens. To produce a signal, for example, a ball is sampled at random from all the balls with the specified meaning. When a new exemplar is stored, a single new ball is added to the urn. Likewise, a deletion removes a single token. As such, this minimal framework employs stochastic production and reception. All other mechanisms are *modifications* of this base framework. As such, WTA production will choose from the most frequent tokens instead of random sampling; broad lateral inhibition deletes a single token of each competing type.

In addition to stochastic production and reception, the basic framework has the following properties which can be assumed to be true of all the following replications unless otherwise stated: The population consists of 10 agents, and we fix the number of meanings and signals at 5 each. There is no use of a restricted context (*C *= *M)*. Agents have no memory limitations or any other form of exemplar deletion such as punishment or lateral inhibition. We employ a closed‐group population dynamic unless otherwise stated. When the gradual replacement population dynamic is used, each new agent interacts with the existing population exactly 35 times.^5^

Before continuing, we must point out that we have made a number of necessarily arbitrary decisions about several parameters. For example, fixing the number of agents at 10 is problematic on at least two levels. The first is in terms of model comparison: In the studies we replicate here, populations range in size between two and ten thousand. Second, and perhaps more important, actual human populations are rarely limited to 10 individuals: We need to know whether the results we present here *scale up* in a reasonable way. Similar concerns can be raised over the choice to model populations as being fully connected (every agent can be called upon to communicate with every other agent) and to limit the number of both signals and meanings to five. The number of signals, for example, can be as low as two in the classic signaling Game of Lewis (1969) or be practically unbounded in a more accurate reflection of human communication (Steels & Loetzsch, 2012).

This being the case, we have opted to set these parameters at relatively small values. The main benefit of this trade‐off is computational efficiency: We are able to run very large numbers of simulations, and this allows us to examine the *aggregate behavior* of any given configuration. That said, the question of scaling must be addressed. Due to the computational costs of running a large number of simulations, we are unable to provide exhaustive results for much larger numbers of agents, signals and meanings. However, results from smaller numbers of runs (see Section 5) indicate that the required number of interaction to lead to optimal signaling appears to increase linearly with the size of the population,^6^ and quadratic growth with the number of signals and meanings. As such, the results presented here remain reasonable for, as an example, populations of 1,000 agents negotiating signaling systems with 100 meanings and signals. We will return to issues of how our framework can address these and other simplifications in the discussion in Section 5, in particular the fully connected network governing agent interactions and the uniform distribution of meanings.

## 4 Results: Exact and minimal replications

In each replication, our aim is twofold. The first is to confirm that our exemplar framework can replicate the original results. To this end, we include all mechanisms from the source paper. Second, and as our principal focus, we want to determine which of those mechanisms are responsible for leading to the reliable development of optimal signaling. This is the role of the basic framework: First, we add the base interaction of each model type—that is, the reinforcement dynamic, the feedback dynamic, or the observational learning dynamic as described in Section 3.1.4. We then test whether this alone leads to reliable optimality. If this is not the case, features from the original models are then added in, individually at first. For those individual features which do not individually lead to optimality, we then investigate whether combinations of those features do.

The purpose of this methodology is to chart out the full range of interactions between the different models and features. In the subsequent section, we provide an overview which argues that despite the initially bewildering range of design choices, they can all be seen as variations on three basic underlying themes. These three mechanisms are the creation and transfer of referential information, a form of information loss, and a bias against ambiguity.

### 4.1 Reinforcement models

We first replicate a basic reinforcement model using Roth–Erev learning as described in Skyrms (2010), before including mechanisms of (a) negative feedback and (b) forgetting (following Barrett & Zollman, 2009), and finally, as an exploratory measure, (c) gradual turnover. The basic reinforcement models require that one idiosyncratic feature is added to the basic framework, *initialization*: All agents begin with one exemplar of each possible association. This avoids a *lock‐in* effect particular to reinforcement models, where agents will only ever produce the first successful signal associated with any given meaning (the result of this is that two agents who had initial success with different signal/meaning associations can never align with each other).

**Figure 4:**
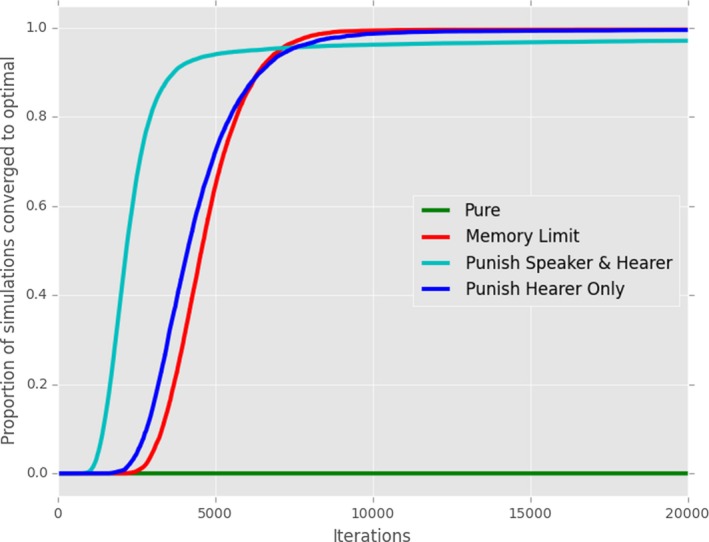
Replication of classic reinforcement learning models. Here, we show the proportion of 10,000 simulations which had converged to an optimal communication system after a given number of iterations. Results are plotted for the pure reinforcement model (“Pure”), pure reinforcement with a memory limit of 35 exemplars (“Memory Limit”), pure reinforcement with punishment for both speaker and hearer (“Punish Hearer & Speaker”), or for the hearer only (“Punish Hearer Only”).

**Figure 5:**
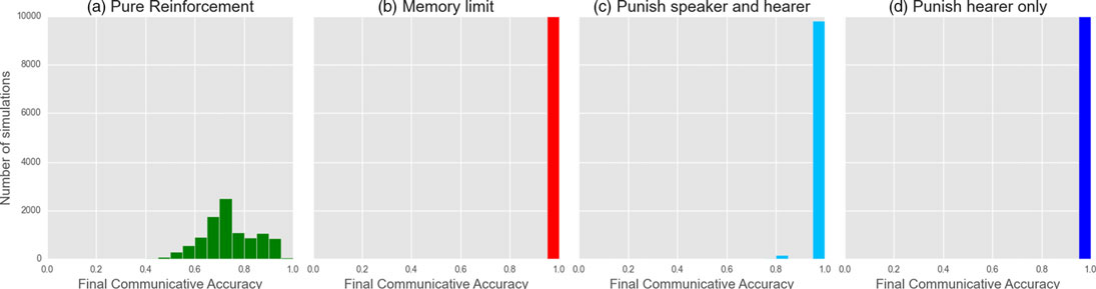
Reinforcement models: comparing (a) pure reinforcement learning with the effects of (b) enforcing a memory limit of 35 exemplars or punishing failed associations for (c) both speaker and hearer or (d) only hearer. Figures indicate the final, stable distribution of communicative accuracy scores for the 10,000 simulations shown in Fig. 4 after 20,000 interactions.

Our replication of Skyrms (2010), which we refer to as the *Pure reinforcement model*, uses the basic framework with the reinforcement feedback dynamic described in Section 3.1.4: After each successful interaction, both hearer and speaker update their exemplar store with the meaning‐signal pair produced by the speaker. Following Barrett (2006), we then add memory limitations and punishment. Results of this set of simulations are shown in Figs. 4 and 5.^7^

Our replication reproduces the key features of the reinforcement models. Pure reinforcement learning as in Skyrms (2010) does not lead to optimality—no runs converge to optimal signaling. Fig. 4 indicates the proportion of 10,000 simulations that have converged after a given number of iterations. Similarly, Fig. 5a displays the final stable distribution of communicative accuracy scores for the same set of simulations. Our normal criterion for optimality is that CA = 1: even when a looser criterion is used (e.g., CA = 0.95), the number of populations which converge is small.

Our first addition to the pure reinforcement model is a *memory limit* of 35 exemplars (Fig. 5b). We confirm that, as shown by Barrett (2006), this reliably leads to optimality; furthermore (not shown), the time taken to reach optimality reduces as the size of memory reduces unless the memory limit is too low to permit a stable set of stored exemplars (e.g., with 5 meanings and 5 signals, once the memory drops much below 25 exemplars). Moving to the second mechanism, *punishment*, we replicate the result from Barrett (2006). When punishment is employed by both speaker and hearer, this usually (but not always) leads to optimality (Fig. 5c). However, as can be seen in Figs. 4 and 5d, if only the hearer modifies his or her exemplar store after each interaction by reinforcement or punishment, optimal signaling reliably develops.

**Figure 6:**
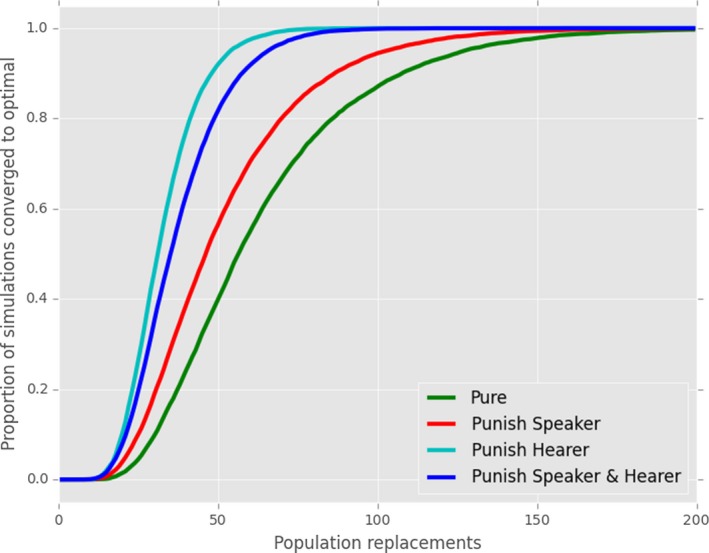
(Modification of Barrett, 2006) Gradual population replacement using pure reinforcement, punishment of speaker only, hearer only, or both speaker and hearer.

Finally, we provide the novel result (Fig. 6) that a third mechanism—gradual population turnover, rather than a closed group—also reliably leads to the emergence of optimal signaling: Under this population model, different types of feedback and updating merely impact on the time taken to convergence. This suggests that limited memory and negative reinforcement are not the only mechanisms that can lead to convergence in a reinforcement model—essentially any process that leads to the removal of stored exemplars (either targeted removal, as is the case of negative reinforcement; random removal, in the case of a memory limit; or wholesale removal, as effected by replacement of individuals) leads to the emergence of optimal signaling within the framework of the reinforcement paradigm.

### 4.2 Naming game models

First, we replicate the Naming Game model of Steels and Loetzsch (2012) using the exemplar framework. The original model Naming Game model is essentially a reinforcement model with a number of added features. Core additions are as follows: (a) full interpretive information is provided after every interaction (i.e., the hearer “points” at its interpretation); (b) referential information is provided after every interaction, although the quality of this referential information depends on the success of the interaction (full referential information is provided after successful interpretation, but only partial referential information otherwise—that is, after the hearer “points” to its interpretation, the speaker says yes or no); (c) lateral inhibition of homonyms and synonyms after storing a new exemplar (see Section 3.1.3); (d) punishment of speaker and hearer after failed communication; (e) WTA rather than stochastic production and reception. Note that our replication differs from the original by limiting the number of signals to 5 and including no restricted context (*c *= |*M|*).

**Figure 7:**
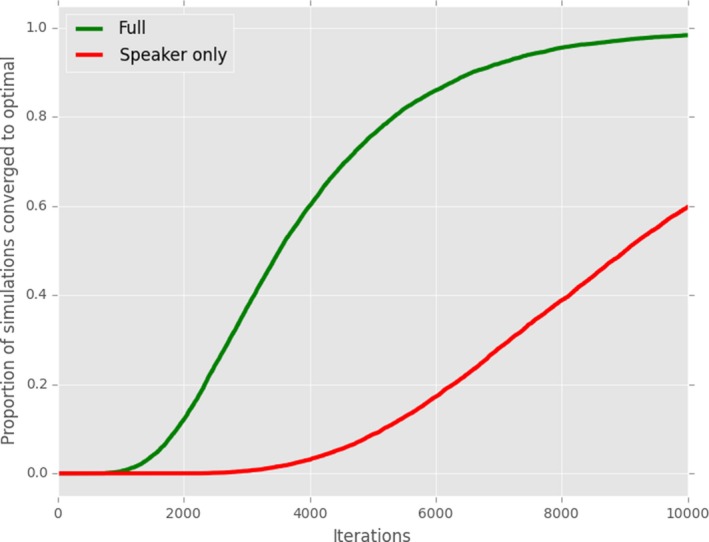
Replication of the Naming Game results of Steels and Loetzsch (2012) and Baronchelli (2010), showing a replication of the full Naming Game (“Full”). We also replicate Baronchelli's results (with a model equivalent to the full Naming Game model, minus referential information), showing that when only the speaker learns from each interaction (“Speaker Only”), convergence is significantly slower than when both Speaker and Hearer learn from each interaction.

As can be seen in Fig. 7, we replicate the original Naming Game result, namely that optimal communication reliably emerges. Furthermore, we replicate the finding of Baronchelli (2010) that when only referential information is provided (i.e., the hearer does not identify their interpretation), whether speaker or hearer updates his or her exemplar store after interaction impacts on time to convergence: Hearer modification leads to rapid convergence, convergence under speaker‐only modification is far slower. This result suggests that interpretative information (the hearer “pointing” to his interpretation) is not required for the emergence of optimal signaling in the Naming Game, assuming the presence of referential information—since referential information is provided in Observational Learning models, but interpretive information is not, this removes one source of difference between these models.

We are now in a position to explore the mechanisms of the model in isolation. As there are actually two feedback dynamics (Yes/No and Referential Feedback), both were investigated using the basic framework.

**Figure 8:**
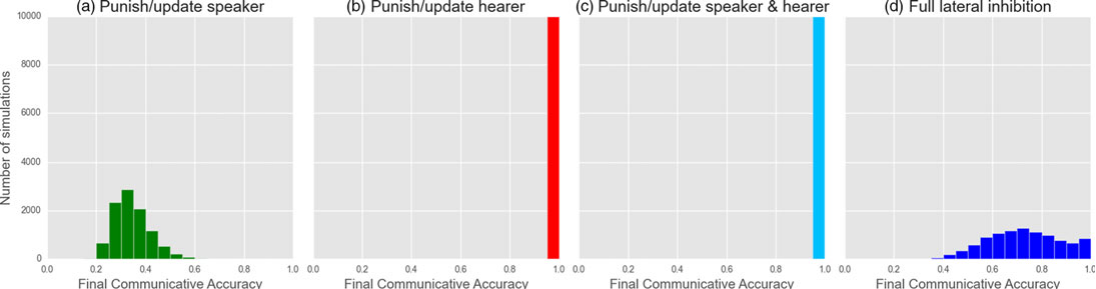
Yes/No Feedback models: investigating the effects of different forms of punishment (a–c) and the application of lateral inhibition of both homonyms and synonyms for both speaker and hearer (d). Figures indicate the final, stable distribution of communicative accuracy scores for 10,000 simulations after 20,000 interactions.

As discussed in Section 2.2.1, Yes/No Feedback is formally identical to reinforcement learning except for one factor: With Yes/No Feedback, the speaker has access to information about the hearer's interpretation. The original feedback model of Steels and Loetzsch (2012) uses a punishment mechanism which has two features: The first is the same as “negative reinforcement” in Barrett (2006)—the speaker punishes the association it used and failed with. The second, which is only available in this model, is that the speaker also punishes the *hearer's* failed association. In Section 4.1, we saw that punishing the hearer after failed communication leads to reliable optimality, while punishing the speaker does not always do so (although it usually does). In the case of Yes/No Feedback, however, we can see in Fig. 8a that when speakers *only* use interpretive information as the basis for update and punishment—that is, when they only punish the hearer's failed association—populations do not reliably develop signaling systems. As shown in Figs. 5b and c, restoring referential information does reliably leads to optimality. However, the importance of the punishment dynamic is illustrated by Fig. 5d, in which we can see that, for Yes/No Feedback *without* any punishment, lateral inhibition of homonyms and synonyms does not reliably lead to optimal signaling.

We can now look at the other feedback dynamic. While punishment is an effective strategy for Yes/No Feedback, it is not effective for Referential Feedback (not shown). Speaker‐only learning and punishment only rarely leads to optimal signaling.^8^

**Figure 9:**
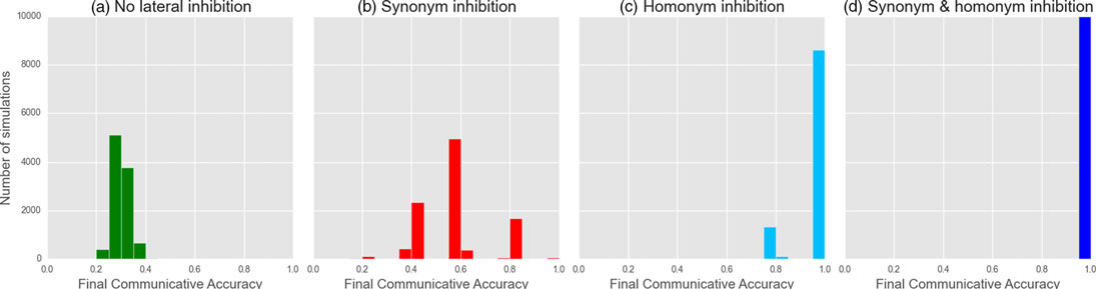
Referential Feedback models: investigating the effects of no lateral inhibition (a), only synonyms (b), only homonyms (c) and both (d). Figures indicate the final, stable distribution of communicative accuracy scores for 10,000 simulations after 10,000 interactions.

Turning to Referential Feedback, the lateral inhibition mechanic (Fig. 9) proves to play a more significant role; specifically, synonyms and homonyms must always be inhibited in order for convergence to occur. A further observation (not shown here) is that the importance of inhibiting synonyms depends on who learns in an interaction. When both speaker and hearer are modified after an interaction, both homonyms and synonyms must also be inhibited; when only the hearer learns, only homonyms must be inhibited.

In summary, the different types of feedback—neither of which leads to optimality by themselves—require different additional mechanisms to do so. As Yes/No Feedback is a modified type of reinforcement learning, it is no surprise that punishment is a successful strategy. What is surprising, however, is that when *only* interpretive feedback (which is the only difference between this model and basic reinforcement with punishment) is used, it never produces a signaling system. Also unexpected is that lateral inhibition does not consistently lead to optimality for Yes/No feedback, while it is highly effective in conjunction with Referential Feedback. In contrast, we see the restricted circumstances in which punishment reliably leads to optimality for Referential Feedback (hearer‐only learning, WTA production). In the final analysis, none of the mechanisms of the Naming Game are unnecessary (except interpretive information for the purposes of punishment). Instead, the model contains a suite of mechanisms, each most effective when applied to the two types of feedback dynamic incorporated into the original model.

### 4.3 Observational learning: Biased learning

Smith (2002) models agents as (*|M|×|S|*) associative networks and explores the consequences of a range of weight‐updating procedures on these networks. In doing so, he identifies two crucial features on which learning rules vary: presence or absence of biases against synonyms and homonyms. Our minimal replication allows us to manipulate these same biases, by enforcing different types of lateral inhibition. Employing a closed‐group dynamic instead of the original gradual turnover, we compare the effects of these manipulations, as well as the role of WTA production/reception (as used in the original) and stochastic production/reception. As this is an observational learning model, only hearers are updated after any interaction, and full referential information is provided. Before proceeding, we point out the similarities between the Referential Feedback model of the previous section and the Biased learning model. The first difference is that the original biased learning model uses gradual turnover instead of closed groups. Second is the provision of information: Both Observational Learning and Referential Feedback guarantee the transmission of referential information, but the dynamic of referential feedback also includes interpretive information. However, we have shown in Section 4.2 that utilizing this information (by punishing failed associations) is not always an effective strategy when referential information is guaranteed.

**Figure 10:**
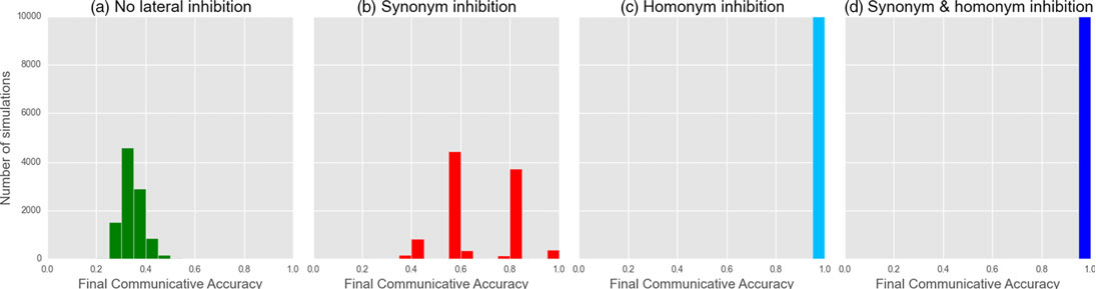
Biased learner models: investigating the effects of no lateral inhibition (a), only synonyms (b), only homonyms (c) and both (d). Figures indicate the final, stable distribution of communicative accuracy scores for 10,000 simulations after 10,000 interactions.

Using the basic framework, we are able to replicate the results of the original (Fig. 10). The emergence of optimal signaling is dependent on learners employing the right type of lateral inhibition; critically, as seen for the Naming Game, agents must *inhibit homonyms*. Again, adding inhibition of synonyms has no additional effect, while inhibiting synonyms alone does not reliably lead to optimal signaling, and optimality never occurs when lateral inhibition is removed altogether. In further tests comparing WTA and stochastic production/reception, no difference was found apart from slightly faster convergence for WTA. This once again suggests that this difference between Smith's model and the Referential Feedback version of the Naming Game described in the previous section is superficial only.

### 4.4 Observational Learning models: Obverter models

Our replication of Oliphant and Batali (1996) uses the Observational Learning version of the basic framework, but also employs *obverter weighting* as described in Section 3.1.1. In line with the observational learning paradigm (Section 3.1.4), only hearers are updated after any interaction. Features of the original model which are not part of our basic framework are (a) WTA production/reception rather than stochastic and (b) gradual replacement population instead of a closed‐group populations. We will examine the effects of these mechanisms, as well as the effects of adding a memory limit. As the effects of lateral inhibition on observational learning models have been fully explored in Section 4.3, none of our replications include this mechanism.

**Figure 11:**
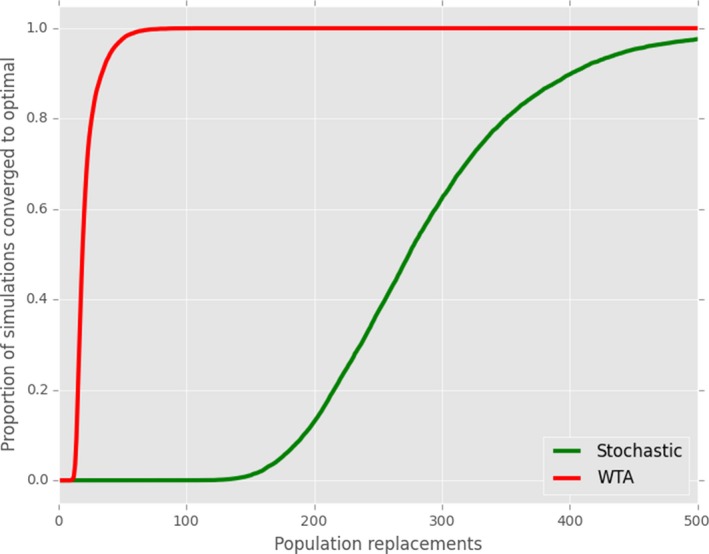
Obverter production/reception, with gradual population replacement, and stochastic or WTA production/reception.

**Figure 12:**
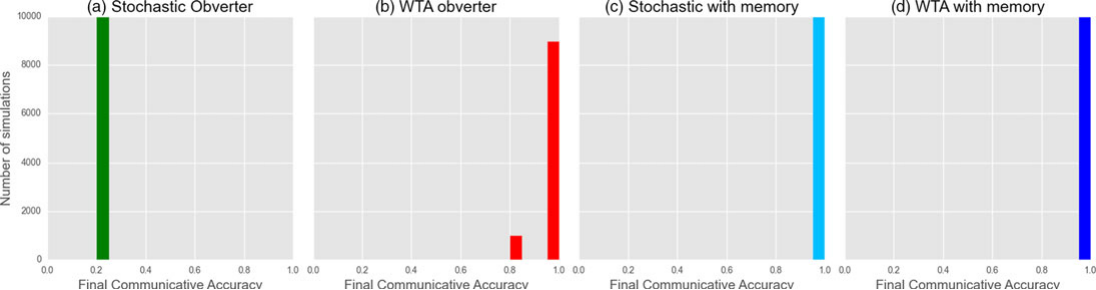
Obverter learner models: investigating the effects of WTA production with and without a memory limited to 35 exemplars (a, b), and stochastic production with and without a memory limited to 35 exemplars (c, d). Figures indicate the final, stable distribution of communicative accuracy scores for 10,000 simulations after 20,000 interactions.

We are able to replicate the original result of Oliphant and Batali (1996): A minimal replication employing gradual population turnover reliably leads to the emergence of optimal signaling when WTA production/reception is used. When stochastic production/reception is used, we still have guaranteed optimality, albeit over much longer time‐scales (Fig. 11). Results for closed groups are strikingly different (Fig. 12). In particular, optimal systems do not reliably emerge unless a memory limit (here, a maximum of 35 tokens) is added to the model.

We see here an strong parallel with the results for Reinforcement Learning. Despite the presence of a *systemic* bias toward functional communication, both strategies are ineffective without the presence of some form of information loss, whether that is via intergenerational transmission or explicit memory loss.

## 5 Minimal requirements for optimal signaling

**Figure 13:**
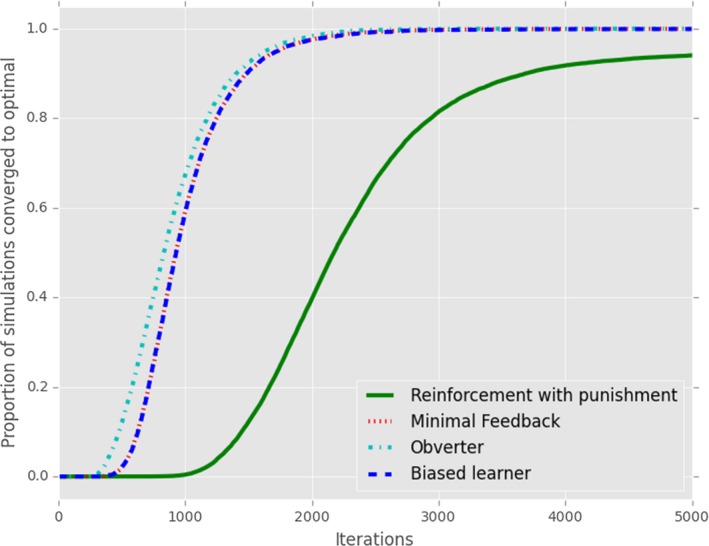
*Model Comparison*: Comparison of the different models instantiated using the basic framework (stochastic production and reception, closed‐group) of Reinforcement (with punishment, following [Barrett, 2006]), a minimal version of the Naming Game using only Referential Feedback, no punishment inhibition of homonyms and hearer‐only update (Steels & Loetzsch, 2012), Obverters with a limited memory of 35 exemplars (Oliphant & Batali, 1996), and Biased learners with inhibition of homonyms (Smith, 2002).

Using our minimal exemplar framework we were able to replicate the results of all the original models. As suspected, the internal representations of agents (network/associations/urn models, etc.) are not a factor behind the development of signaling; all that is necessary is a way to model agents who can capture associations between meanings and signals. Indeed, given the appropriate parameter settings, the replications using the basic framework produce results which are strikingly similar or identical (Fig. 13) when we compare a version of the Naming Game with hearer‐only learning and only Referential Feedback with the Biased Learning model (both using lateral inhibition of homonyms).

Beyond this, what conclusions can we draw? First, passing *referential information* (as described in Section 3.1.4) from speakers to hearers is almost always essential. In the case of Observational Learning and classic variants of the Naming Game where referential information is provided, full referential information is always provided to the hearer. This allows them to know with certainty which meaning the speaker intended to convey; the only difference between the referential feedback models and observational learning models is that, in the latter, the topic is immediately provided. In Naming Game models with referential feedback, it is provided after the signal has been sent. However, as evidenced by the reinforcement and Naming Game models with Yes/No Feedback only, it is enough for referential information to only be provided sometimes. More interestingly, reliable interpretive information (i.e., the hearer signaling its interpreted meaning to the speaker) does not appear to play a significant role, as can be seen in Naming Game models with Yes/No feedback where only the speaker learns from each interaction.

Second, *information loss* about previously stored associations must be present in some form. In reinforcement learning and the obverter instantiations of the observational learning model, this can be due to deletion, limited memory, or population turnover. In the case of Naming Game and Biased Learning models, this can be achieved via lateral inhibition.

In the Naming Game and Biased Learning models, lateral inhibition of homonyms also serves to provide a *bias against ambiguity*. This bias seems to be intrinsic to the reinforcement model and obverter models; we discuss this in greater detail below.

**Figure 14:**
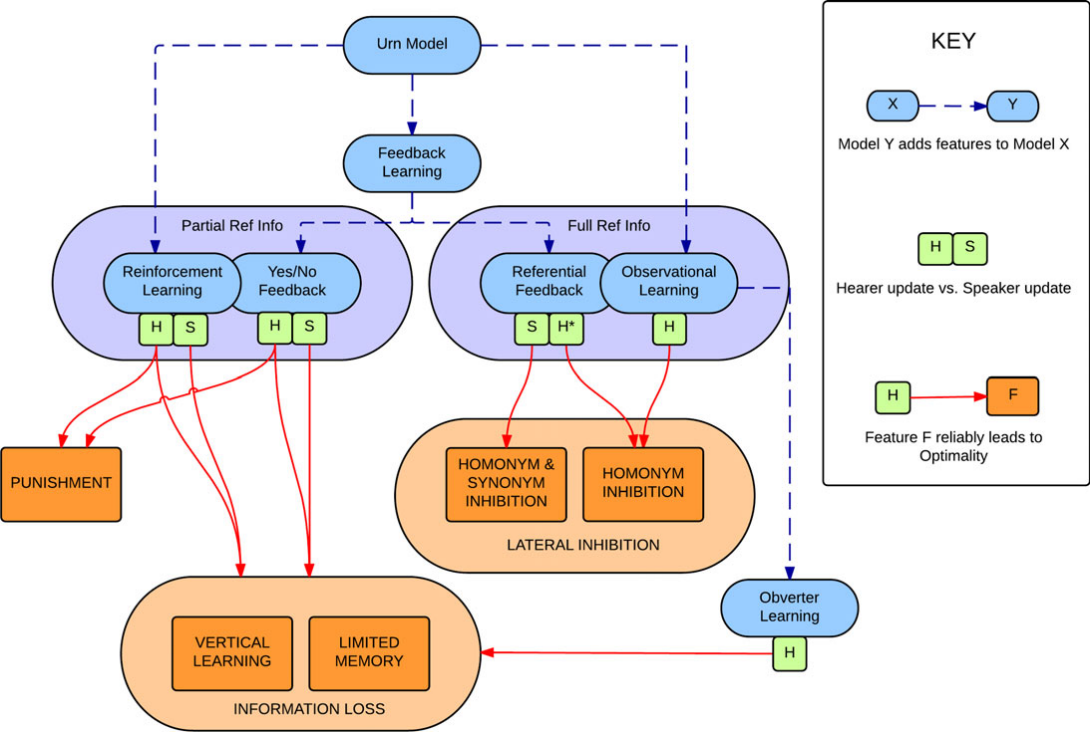
An overview of how the different models (in blue) relate to each other in terms of the availability of referential information (guaranteeing either partial or full referential information), and the model mechanisms (in orange) which reliably lead those models to optimal signaling. Vertical learning and limited memory are grouped together as being types of information loss; the two forms of lateral inhibition are also grouped together. Green squares represent the impact of Speaker and Hearer update, respectively; for example, only hearer‐directed punishment drives reinforcement to optimality, while both hearer and speaker‐directed punishment are effective for Yes/No feedback models. For Referential Feedback, the * denotes the fact that a specific configuration (hearer‐directed punishment with WTA production) does lead to optimality, but this is not otherwise indicated.

A summary of these findings can be seen in Fig. 14, which indicates how the different models relate to each other in terms of the basic framework, and which mechanisms are required for each model to reliably develop optimal signaling.

### 5.1 Referential information

One requirement for optimal communication is conventionality: A population agrees on how signals map to meanings. The problem is that while signals are overt, meanings are not. If only signals passed between agents, arriving at a convention for how meanings and signals are associated would be impossible; a mechanism for sharing this referential information therefore must exist. Fig. 3 in Section 3.1.4 shows the two types of information that can be made available in any interaction between agents. Referential information details how a speaker provides an intended referent; interpretive information pertains to how a hearer interprets a given signal. How does this apply in the models above?

In Observational Learning models, full referential information is always provided (guaranteeing transfer of meaning as well as signal between speaker and hearer), and interpretive information is never provided. In basic reinforcement learning, the environment provides full referential information and interpretive information after communicative success, and partial information of both types after failure (although in the latter case this information is only utilized when deletion of exemplars after unsuccessful communication is employed, i.e., punishment). The Naming Game model of Steels and Loetzsch (2012) involves two flavors of feedback. Referential Feedback ensures that, whether a given interaction has been a communicative success or a failure, full referential and interpretive information is always provided. With Yes/No Feedback, full interpretive information is always provided (as the hearer always indicates their interpretation), and the amount of referential information provided depends on the success of the interaction—full referential information is only provided after success (as the speaker confirms the hearer's interpretation), whereas partial referential information is provided after failure (some other member of the context must have been the intended meaning). Note again that this is almost equivalent to Reinforcement learning except for the fact that full interpretive information is always provided via the hearer's pointing action.

This analysis in terms of information flow rather than agent interaction clarifies a number of issues. First, the reason Reinforcement models and Yes/No‐only variants of the Naming Game are slower to converge is simply that full referential information is not as frequently provided (i.e., only after successful communication, rather than after every interaction). Second, the *identical behavior* of the minimal instantiations of the Naming Game and biased Observational Learning models shown in Fig. 13 is no surprise; in both—despite the different sequencing of information transfer—hearers are always supplied with full referential information about the speaker's signaling behavior. Finally, while both Naming Game and Reinforcement models are often implemented so as to provide interpretive information, this is not a *necessary* component for the development of optimal signaling, as shown in Fig. 7 in Section 4.2, in agreement with the conclusions of Baronchelli (2010). The only exception to this is versions of Reinforcement and Feedback learning where only speakers learn; in this case, the guaranteed transfer of interpretive information proves to be critical in ensuring optimality.

Crucially, conventionality is established in a population whenever speakers propagate referential information to hearers. This is backed up by a mathematical result from Xue (2006). Xue employs populations of interacting Pólya urns; this is essentially a generalized version of our basic framework described in Section 3.2. Xue shows that whenever information is passed from speaker to hearer with positive probability, a state of conformity will always result. This, then, is the fundamental role of the various channels of information transfer between speaker and hearer: ensuring conformity primarily via the transfer of referential information about the topic from signalers to hearers.

### 5.2 Bias against ambiguity

With the spread of referential information, we can expect the development of conventionality in a population. Unless other pressures are at play, however, these conventions are unlikely to be optimal, as illustrated by the “imitation learner” of Oliphant and Batali (1996) and the “maintainers” of Smith (2002). A pressure toward optimality is created in various ways: when only successful associations are strengthened, when obverter weighting is used, or when there is inhibition of homonyms. What do these mechanisms have in common?

Our definition of optimality requires that an unambiguous signal exists for each meaning and that all signals are unambiguous. Ambiguity indicates the presence of some degree of homonymy: It is no surprise that the explicit deletion of homonyms leads to an unambiguous system. This *lateral inhibition* of homonyms is the pressure that drives optimality in biased Observational Learning (Smith, 2002) and Naming Game (Steels & Loetzsch, 2012) models.

In Reinforcement models, reinforcement strengthens successful associations, therefore preferentially increasing the weight of less ambiguous associations for the hearer. That association is then more likely to be used again, by the hearer when later acting as a speaker. This *rich‐get‐richer* process provides the necessary pressure against ambiguity. Obverters also have an inherent bias against ambiguity, manifested by picking the least ambiguous signals. However, in the absence of some form of memory loss, this bias is either never strong enough (with stochastic production) or not reliably so (WTA) to lead to guaranteed optimality.

We draw attention to the fact that the bias against ambiguity has multiple possible interpretations. In Reinforcement learning the bias in incorporated in both the environment (which provides a payoff when states and acts are matched), and the ability to recognize that payoff—or, when punishment is employed, the lack of a payoff. Similarly, in the nearly equivalent Naming Game model with Yes/No Feedback, information about success is established through *interaction*. In the Naming Game with referential feedback, as with observational learning models, the guarantee of referential information requires an internal bias in the form of lateral inhibition of homonyms. More sophisticated still (but mechanistically identical) is the “rational” approach of the obverter Observational Learning model.

As a final remark, we point out a parallel between this pressure against ambiguity and the “amplifying function” described in De Vylder and Tuyls (2006) (outlined here in Section 2.2), which works to eliminate synonyms and guarantees convergence in the homonymy‐free naming game. The true function of the bias against ambiguity is seen in the way input meaning/signal distributions map to output meaning/signal distributions, that is, from what an agent learns, to what an agent produces. While De Vylder's amplifying function acts to always privilege more common variants in the mapping from input to output, the required bias against ambiguity applies slightly differently. Instead, the bias ensures that the *least ambiguous* variants be amplified via repeated cycles of learning and production.

### 5.3 Information loss

Section 4.1 highlighted the necessity of *information loss* in the development of optimal communication. This can take several forms: simple forgetting through limited memory size, the sampling effects of gradual replacement of members of the population, or more targeted processes of deletion and lateral inhibition.

Why should information loss be beneficial, rather than a hindrance? In all models, the initial state of the population is highly disorganized: Individual agents have maximally ambiguous meaning/signal associations and are driven in many different, mutually incompatible directions as a result of their early interactions. If an optimal signaling system is to be established, the influence of these early disorganized states must be eliminated. In standard Roth–Erev learning (and hence also exemplar learning), learners have an effectively infinite memory and place an equal weight on all observations. Because the effect of new information is proportional to the count of previous observations, the *learning rate* steadily decreases over time. In the case of classic instantiations of the Reinforcement model—and also in obverter Observational Learning without population turnover or forgetting—we observe a slowing effect. In the long term, populations are trapped into non‐optimal pooling equilibria. The only way to avoid this is some form of information loss. It provides a “plasticity” whereby non‐optimal states can always be escaped.

As in the previous section, we note that the mechanisms which lead to information loss can have very different interpretations and apparent functions. However, they all share two key properties: (a) new information is privileged over old, and (b) the chance that a particular association “survives” (either within an individual or within the population as a whole) is proportional to its relative frequency, making possible the stability of frequent associations against noisy loss.

### 5.4 Comment: Simplifications and extensions

Our framework involves a number of simplifications. To expand on our comments in Section 3.2, increasing the population size appears to result in roughly linear growth in the number of interactions required for convergence, but quadratic growth when increasing the number of meanings and signals. To measure the effect of increasing the population, we took the average time to convergence over 10 simulations for the original population size. We then successively doubled that size to attain values for populations of 10, 20, 40, 80, 160, 320, 640, and 1,280. The time to convergence was found to roughly double for each doubling in population. A similar method was used for meanings and signals, with 5, 10, 20, 40, 80, and 160 tested. In this case, each doubling resulted in a roughly fourfold increase in time to convergence. However, due to the time constraints imposed by computational limitations, we are not able to make any strong claims on this basis, particularly in light of the fact that Barr (2004) observed something more like logarithmic growth when he increased the number of agents in his simulations. In any case, the linear or slower growth resulting from increased population sizes seems less problematic than the quadratic increase which occurs when more meanings and signals are used. The emergence of novel sign languages such as NSL involves large signal and meanings inventories. These results suggest that this process might necessarily be piecemeal, first establishing small number of conventional meaning‐signal mappings and then expanding. It would be an interesting direction for future research to empirically verify whether this is indeed the case.

Moving away from matters of simple scaling, the fully connected populations used in the model are quite unlike the social structures found in actual human societies, which tend to exhibit the “small‐world” property identified by Milgram (1967). This is a very rich area of study and impossible to treat thoroughly here, but we draw attention to the possibility of an interaction between the agent model and the network type; indeed, Barr (2004) shows that one very simple strategy (*stay/switch*, described in Section 2.2) is ineffective in fully connected populations but seemingly optimal in more sparsely connected networks. In some further preliminary work, we have investigated the effects of placing agents on small‐world networks and also very sparsely connected lattice networks. For small‐world networks, it appears that the short average path‐length between any two agents leads to no great divergence from our observations above. For lattice networks, however, where any two agents can be separated by a significant number of intermediaries, we found that the global emergence of a single set of optimal signaling conventions was not guaranteed for the majority of simulation runs even under parameter setting which lead to convergence in fully connected populations; rather, populations converge on a series of local optimal conventions (as also seen in Smith, 2003). This certainly warrants further investigation, but we are encouraged by the fact that the more realistic network structures do not appear to conflict with our proposed general requirements.

Finally, as observed by Zipf (1936), words in natural languages tend to roughly follow a power law distribution; that is, the frequency *f* (*w*) of a word scales more or less according to its frequency *r* rank *r*, so that $f\left(w\right)\propto \frac{1}{r}$. We amended our model so that the presentation of meanings had such a distribution, first for the case of 5 meanings and signals, and then for 50 meanings and signals, and compared the resulting number of iterations to convergence with those for uniform meaning distributions. These investigations found a distinct effect whereby the power‐law distributions led to slower convergence, but within the same order of magnitude (*≈*1.5 times as long for 5 signals and meanings, and *≈*3 times as long for 50). As such, we are content that—at least in this case—our results are robust to manipulations of meaning frequency.

## 6 Conclusions

To reiterate the remarks of Section 5, we argue that the necessary requirements leading to the reliable development of optimal signaling conventions in populations of interacting agents are as follows:

1. A way of propagating *referential information*
2. The presence of a *bias* against ambiguity/homonymy
3. Some form of *information loss*

One way to look at these requirements is as a solution at Marr's *computational* level of analysis (Marr & Poggio, 1976). There are, however, multiple solutions at the *representational* level—presumably more than the four we have surveyed here. With this in mind, what can we say about which strategies are actually employed by humans, whether in naturalistic or experimental settings? At this point, it is worth re‐examining two of the requirements—reference and bias—as there is a discernibly common pattern in how they are treated.

In contrast to Lewis's (1969) proposal that common ground must play a role in the establishment of optimal signaling, none of the theories here require global knowledge beyond agreement on a set of shared meanings and signals. Where they differ is in how reference is established through individual interactions. Reinforcement learning requires environmental cues to create reference where none existed before; models in the Naming Game framework assume the existence of referential meaning but concern themselves with how pairs create shared reference; in observational learning models, the salience of reference is assumed. In all cases, referential information is shared during interaction.^9^ The different roles of reference in these models can be construed as involving increasing degrees of cognitive sophistication: first environmental stimulus, then explicitly negotiated, and finally implicitly available. Humans use all these strategies (Ashby, Maddox, & Bohil, 2002; Fay, Arbib, & Garrod, 2013; Scott‐Phillips, Kirby, & Ritchie, 2009). Reinforcement learning is arguably the simplest account here in cognitive terms.

A similar trend can be seen with the bias against ambiguity. Reinforcement learning requires only recognition of variability in stimuli: *Operant conditioning* (surveyed in Staddon & Cerutti, 2003) has long been established as a common animal behavior. Inhibition of homonymy is a type of *mutual exclusivity bias* (Markman & Wachtel, 1988), and more complex pragmatic inference resembling obverter learning has been experimentally observed in language games by Frank and Goodman (2012). Again, reinforcement learning appears to present the simplest strategy, while not necessarily the most efficient one.

Modern humans can utilize all the cognitive abilities described above. Which, then, are involved in the self‐organization of functional communication systems we see in the wild (e.g., in the emergence of homesign or indigenous sign languages such as NSL or ABSL and in the lab (e.g., in studies on experimental semiotics)? Presumably, there must be some interplay between the individual task demands of the particular communicative setting—whether naturalistic or experimental—and the cognitive expenditure which is required. It is very likely, for example, that certain communicative settings will favor a particular strategy where others favor a different one, perhaps selected depending on the type and quality of feedback available. We suggest that this may be a fruitful line of enquiry for future experimental work.

What we hope our comparative approach has shown is the *multiple realizability* of behavior that leads to the emergence of signaling conventions. Compelling evidence for the explanatory role of any particular mechanism (e.g., learning bias or feedback) should not be taken as evidence for that being the *only* explanation. Hopefully, we can instead *integrate* these numerous insights and gain a richer understanding of the tapestry of human communicative behavior.

## Notes

1 This is essentially a type of *conformist frequency‐dependent bias* (Boyd & Richerson, 1985).

2 This is in the case of well‐connected populations: Neural network populations did not arrive at global convergence when they were placed on “small neighborhood” network topologies.

3 The source code for the model can be found at https://github.com/matspike/Cogscisignaling

4 Formally, *T* = {*C* ⊆ P(M)| |*C*| = *c*}.

5 This sufficiently reduces the probability that any given meaning is not sampled.

6 Note, however, that Barr (2004) observed non‐linear, logarithmic growth when using expanded populations.

7 In subsequent figures, we will make use of time‐course graphs when we feel some indication of relative speed of convergence is necessary. The final distribution of communicative accuracy scores will be shown otherwise. We point out that these distributions are *stable* and are not to be taken as intermediate stages which may yet develop optimality.

8 On closer inspection of individual simulation time‐courses, hearer‐only learning and punishment reliably tends toward optimality but almost never reaches it. This is due to the stochastic nature of the basic framework: Because minority meaning/signal associations are less likely to be used by the population, they are also increasingly less likely to be deleted (a type must be used to be unsuccessful). In the specific case of WTA hearer‐only learning and punishment, this effect is avoided. This is the only time when WTA instead of stochastic processes prove to be a critical factor in the development of optimal signaling.

9 There is an issue we have not addressed here, which Smith (2005) refers to as the “signal redundancy paradox”; that is, why signal if meaning can be transferred otherwise, for example, via pointing? There are a number of approaches to this problem, ranging from the observation that signaling, once established, allows for both displaced reference and communication out of the line of sight, to the proposal by Smith (2005) that most meaning transfer must not be explicit but require sophisticated inference. This is very interesting work, but we follow the precedent in much of the earlier work on the evolution of signaling systems by deferring the study of this important question.
